# Halting the Progression of Alzheimer’s Disease: Is the Goal in Sight?

**DOI:** 10.1007/s12264-024-01339-3

**Published:** 2024-12-27

**Authors:** Yu-Juan Jia, Xuan-Yue Wang, Jie Liu, Yan-Jiang Wang, Colin L. Masters, Jun-Hong Guo

**Affiliations:** 1 Department of Neurology, First Affiliated Hospital, Shanxi Medical University, Taiyuan, 030001 China; 2 Department of Neurology and Centre for Clinical Neuroscience, Daping Hospital, Third Military Medical University, Chongqing, 400042 China; 3 Department of Memory Principles, Chongqing Institute for Brain and Intelligence, Chongqing, 401125 China; 4 Department of Neurology and Centre for Clinical Neuroscience, Daping Hospital, Third Military Medical University, Chongqing, 400042 China; 5 Key Laboratory of Ageing and Brain Disease, Chongqing, 400038 China; 6 The Florey Institute, The University of Melbourne, Parkville, VIC 3052 Australia

Alzheimer’s disease (AD) is a slow, progressive neurodegenerative disease with clinical symptoms that typically emerge in the elderly, leading to deterioration of cognitive functions over time. Memory loss is the primary symptom, eventually leading to significant declines in executive and cognitive functions, along with psychiatric and behavioral changes, and alterations in personality. The exact cause of AD remains unclear. Presently, the “Aβ cascade hypothesis” is widely accepted, proposing that the excessive buildup of Aβ initiates the intricate pathological processes of AD. This accumulation precedes clinical signs by 15–20 years and is a pivotal event in AD progression [1].

Aβ, an extracellular peptide, is generated from the amyloid precursor protein (APP) *via* sequential cleavage by β-secretase and γ-secretase. Although APP was initially thought to be predominantly expressed in neurons, recent studies have shown that other cell types, especially oligodendrocytes, also express it and produce Aβ [2]. The most common forms of Aβ are Aβ40 and Aβ42, the latter being more prone to aggregation and therefore more neurotoxic [3]. In addition, N-terminally truncated and pyroglutamate-modified forms of Aβ have also been implicated in AD pathology [4, 5]. Normally, Aβ plays a role in intercellular communication and the maintenance of synaptic plasticity [6]. Under physiological conditions, excess Aβ is primarily cleared from the brain through microglial phagocytosis and the glymphatic system, which involves cerebrospinal fluid and blood circulation [7]. However, in AD patients, these clearance mechanisms become impaired, leading to the accumulation of Aβ. While microglia are essential for Aβ clearance, recent studies have also revealed their involvement in amyloid plaque formation, suggesting a more complex role beyond simple phagocytosis [8]. This dual function of microglia in both seeding and clearing Aβ contributes to the intricate dynamics of AD pathology. As Aβ accumulates, it triggers downstream events such as Tau protein tangles, oxidative stress, and neuronal degeneration, ultimately resulting in brain atrophy and cognitive decline. With advances in understanding the pathological mechanisms of AD, the diagnostic approach has shifted from traditional clinical exclusion to a pathology-based diagnosis centered on Aβ [9]. Consequently, targeted clearance of Aβ has emerged as a key area of research for AD treatment.

**Fig. 1:**
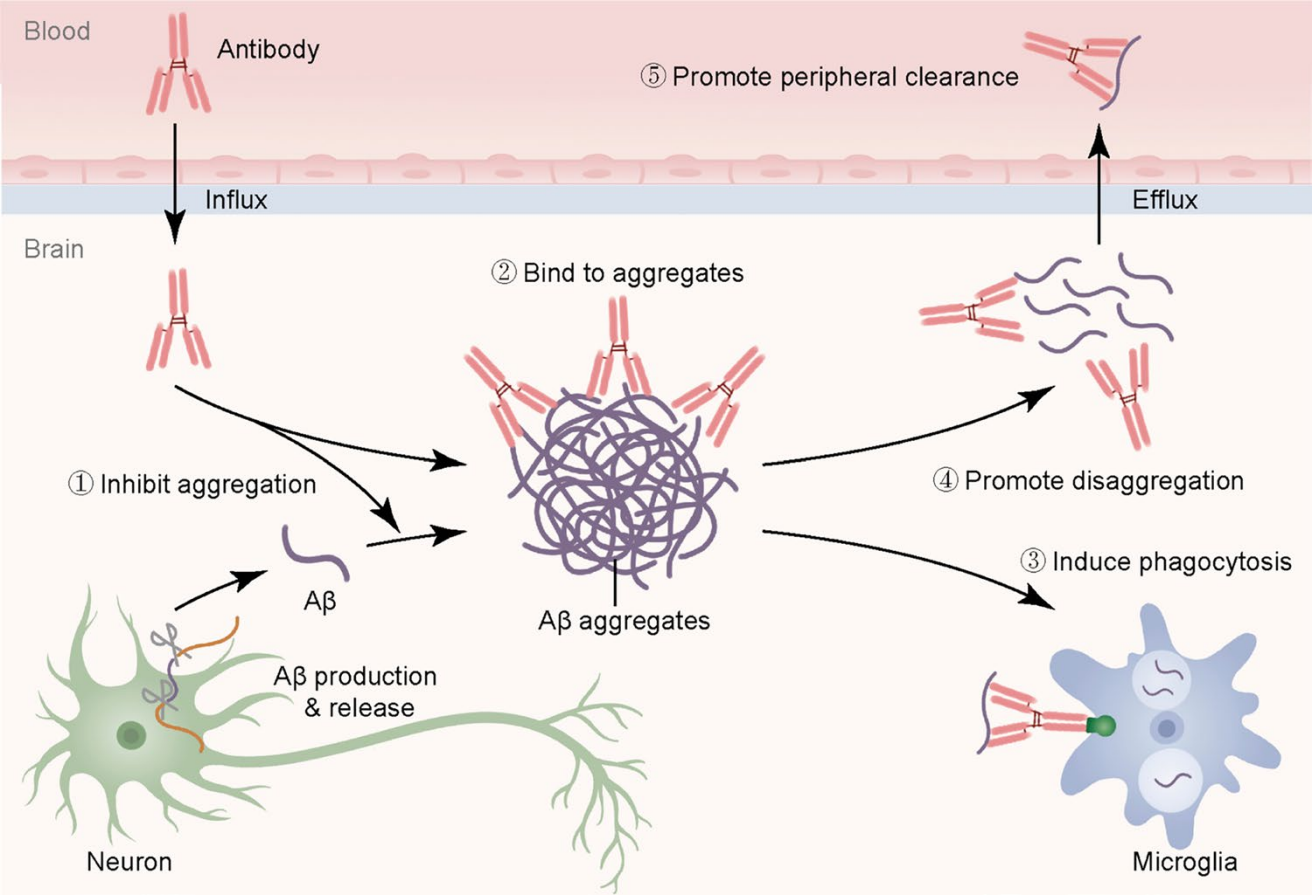
The mechanisms of action of anti-Aβ antibodies. After crossing the blood-brain barrier, Aβ antibodies inhibit the aggregation of Aβ monomers, bind to Aβ aggregates, promote the disaggregation of Aβ aggregates, induce the microglial phagocytosis of Aβ, and enhance the peripheral clearance of Aβ. These combined mechanisms help to reduce Aβ accumulation in the brain, alleviating the pathological burden of AD.

In the 1990s, it was discovered that Aβ antibodies exist in the human body. Subsequent studies confirmed that these antibodies inhibit Aβ aggregation and promote Aβ fiber depolymerization (Fig. 1), ushering in a new era in AD immunotherapy [10]. Dr. Dale Schenk and colleagues first proposed active immunotherapy for AD in 1999, but this was halted due to severe side-effects such as T-cell overactivation and autoimmune encephalitis [11]. To address these issues, passive immunotherapy for AD was suggested, involving direct infusion of Aβ antibodies to clear Aβ from the brain. From 2012 to 2020, four Aβ monoclonal antibodies aimed to enhance cognitive function in AD patients by targeting Aβ removal, but all failed [12–15]. In 2021, the EMERGE phase 3 clinical trial of aducanumab demonstrated its effectiveness in clearing Aβ from patients’ brains and delaying cognitive decline, while the parallel ENGAGE trial did not yield the same results [16]. The FDA analyzed previous Aβ antibody trials and found a direct link between an antibody’s ability to remove Aβ and its capacity to delay cognitive decline. Consequently, the FDA expedited aducanumab’s approval, sparking widespread debate on the efficacy of Aβ antibody therapy. In September 2022, the Clarity phase 3 clinical trial of the lecanemab antibody achieved significant success [17]. After 18 months of treatment, Aβ levels in the brains of patients in the treatment group returned to normal. These patients experienced a 27% reduction in the rate of cognitive decline compared to the placebo group, with CDR-SB (Clinical Dementia Rating—Sum of Boxes) scores improving by -0.45 (*P* <0.01). Some experts view the success of the Clarity trial as validation for targeting Aβ removal in AD treatment. However, others argue that, while statistically significant, the clinical improvement in the trial is not substantial. Furthermore, the serious adverse events reported, such as amyloid-related imaging abnormalities (ARIA) with edema (ARIA-E) or microhemorrhage (ARIA-H), warrant careful consideration, as risking potential death for uncertain benefits should not be taken lightly.

During the dispute, the August 2023 phase 3 clinical trial of the donanemab antibody (TRAILBLAZER-ALZ 2) revealed that almost half of early-stage AD patients experienced no further decline in cognitive function after one year of donanemab treatment [18]. TRAILBLAZER-ALZ 2 was a randomized, double-blind, placebo-controlled study involving 1,182 patients with Mild Cognitive Impairment and Mild Dementia-stage AD. The primary endpoint metric (Integrated Alzheimer’s Disease Rating Scale) indicated that donanemab delays cognitive decline by up to 35%, while a significant secondary endpoint metric (CDR-SB score) shows a 36% delay in cognitive decline after 18 months. Notably, 47% of patients on donanemab do not show clinical progression (defined as no decline in CDR-SB score) after one year of treatment, with 52% achieving complete clearance of Aβ plaques from the brain over the same period. This outcome suggests that disease progression halts nearly half of early AD patients after one year of donanemab treatment. The success of donanemab validates the efficacy of antibody therapy targeting Aβ clearance in AD treatment, demonstrating that adequate clearance of Aβ from the brain effectively slows or even halts disease advancement.

Throughout the extensive history of active and passive immunotherapy, amidst numerous failures and continuous improvements, the overall success of the donanemab antibody has fostered confidence in conquering AD. Moreover, it has provided empirical lessons for further exploration of targeted Aβ immunotherapy. First, the success of lecanemab and donanemab powerfully confirms that targeted Aβ immunotherapy should be administered early in the disease. Notably, one reason donanemab proves more effective than lecanemab may be in applying the severity of Tau accumulation for stratification during patient selection and grouping, rather than relying solely on cognitive level. As is well known, Tau accumulation is closely linked to disease severity [19]. Patients with moderate Tau-PET levels are deemed to be in the early stage with mild cognitive impairment, and the findings have confirmed the significant effect of targeted Aβ clearance. Conversely, patients with high tau-PET {Citation}and the results demonstrate lower efficacy. Similarly, findings from the DIAN (Dominantly Inherited Alzheimer Network) trial also emphasized the critical importance of early intervention [20]. In some carriers of a dominantly inherited AD mutation, amyloid immunotherapy not only prevents tangle formation but also restores memory in those with mild symptoms. These findings underscore the necessity of accurately screening patients in the early stages of AD based on biomarkers. Building on this, the AHEAD 3–45 study is currently testing whether long-term, low-dosage immunotherapy can prevent amyloid accumulation in at-risk individuals, such as those carrying the APOE4/4 allele. This approach aligns with the hypothesis that early and sustained intervention can prevent cognitive decline and minimize the risks of adverse effects, such as ARIA, associated with higher dosages. Though the final results of AHEAD 3–45 are yet to be published, the study represents a critical step toward understanding the long-term benefits of early preventive treatment. Secondly, the phase 3 trial showed that donanemab results in 52% of subjects achieving complete clearance of Aβ plaques from the brain within a year, while 72% achieve complete clearance of Aβ plaques at 18 months [18]. In contrast, the phase 3 clinical trial of lecanemab reported that two-thirds of treated patients had intracerebral Aβ clearance to normal levels (below the positive threshold) after 18 months of treatment, and cognitive decline was effectively delayed [17]. Therefore, the ability of donanemab to halt disease progression may be related to complete clearance of Aβ from the brain. Consequently, adequate and safe clearance of intracerebral Aβ is anticipated to impede disease progression and should be the primary therapeutic target for Aβ clearance. Recently, Eli Lilly has developed another monoclonal antibody named remternetug, which has exhibited a more rapid and potent clearance of plaques. In particular, high doses of remternetug have proven to completely remove intracerebral Aβ plaques in early AD patients after only 12 weeks of treatment. Notably, remternetug has demonstrated good tolerability and safety. Presently, it is undergoing a phase 3 clinical trial, and the outcomes are highly anticipated [21].

Although there has been initial progress in Aβ monotherapy for AD, it is uncertain whether it will be the first AD disease-modifying therapy, a medical intervention that alters the clinical trajectory of the disease. Studies have shown that donanemab effectively clears Aβ from the brain and eliminates cognitive decline in nearly half of AD patients within 1 year. However, the study was conducted over 18 months, making it unclear whether patients would benefit in the long term. Some suggest that it may not be necessary to continue Aβ antibody therapy after 18 months, or that continuing the therapy may not increase the benefit but rather increase the incidence of drug-related adverse events. Longer interventions are needed to clarify the long-term benefits of Aβ antibody therapy. In addition, it is important to carefully monitor the adverse reactions associated with Aβ antibody therapy, specifically ARIA-E and ARIA-H. While most patients experience only transient symptoms, fatal cases have been reported, particularly in ApoE4 carriers and patients at risk of hemorrhage [17, 18]. Therefore, caution must be exercised when administering these drugs to high-risk patients. Further studies are needed to clarify the etiology and mechanisms behind the development of ARIA-E and ARIA-H.

Previous studies have shown that individuals carrying the ApoE4 gene derive little benefit from Aβ antibody therapy and are at a significantly higher risk of developing ARIA-E and ARIA-H compared to non-carriers [17, 18]. It is known that in patients with sporadic AD, ApoE4 is the most relevant risk gene and an important factor in the early age of onset. However, the current Aβ antibody therapy does not improve the disease status of these patients. Therefore, finding alternative therapeutic strategies is imperative. In recent years, some investigators have attempted to remove phosphorylated Tau proteins, but significant results have yet to be obtained [22]. We hypothesize that the deposition of hyperphosphorylated Tau protein is the metabolic fate of microtubule proteins after neuronal degeneration in the later stages of neurodegenerative diseases. Targeting this protein may not yield better therapeutic results for disease intervention. It is known that 95% of AD cases are sporadic, and the root cause lies in the dysfunction of the clearance mechanisms of microglia and the glymphatic system. The current strategy for Aβ antibody therapy is adaptive immunotherapy for intrinsic immune diseases. Therefore, enhancing the functions of both microglia and the glymphatic system for effective Aβ clearance may be fundamental for the treatment of AD [23]. Furthermore, strategies aimed at rejuvenating microglia, such as targeting lipid droplets and replenishing the microglial pool, could play a crucial role in improving Aβ uptake [24]. For individuals at high risk of AD, it is important to implement tertiary prevention measures to maintain homeostasis of the brain environment [25]. This will help to preserve the functional status of microglia and minimize Aβ overproduction and aggregation. In cases where neurons are already experiencing inflammation and degeneration due to Aβ over-deposition, it is crucial to explore effective neuroprotective strategies to restore neuronal function and prevent or even reverse the disease state [26]. Finally, regarding immunotherapy, the DIAN study suggests that initiating treatment in the early, pre-symptomatic stages yields more pronounced effects, not only slowing the progression of Aβ pathology and the emergence of Tau tangles but also significantly delaying cognitive decline.

## Conclusion

Although the success of lecanemab and donanemab confirms that Aβ antibodies are expected to be a disease-modifying strategy for AD treatment, the long-term benefits, the risk of adverse effects, and the degree of patient tolerance still need to be established. On this basis, we need to investigate the pathogenesis of AD and biomarker changes in the early stages of the disease. This will help to accurately identify AD patients in the early pathological stages, select suitable intervention targets, and identify indicators to evaluate the intervention’s effectiveness. Meanwhile, the etiology and risk factors for the occurrence of ARIA should be clarified to minimize the occurrence of serious adverse events. Finally, exploring interventions to enhance microglial phagocytosis and effective neuroprotective strategies are important in defeating AD.
